# Low Handgrip Strength (Possible Sarcopenia) With Insulin Resistance Is Associated With Type 2 Diabetes Mellitus

**DOI:** 10.1210/jendso/bvae016

**Published:** 2024-02-02

**Authors:** Tsubasa Tajima, Hideyoshi Kaga, Yuki Someya, Hiroki Tabata, Hitoshi Naito, Saori Kakehi, Naoaki Ito, Nozomu Yamasaki, Motonori Sato, Satoshi Kadowaki, Daisuke Sugimoto, Yuya Nishida, Ryuzo Kawamori, Hirotaka Watada, Yoshifumi Tamura

**Affiliations:** Department of Metabolism & Endocrinology, Juntendo University, Tokyo, 113-8421, Japan; Department of Metabolism & Endocrinology, Juntendo University, Tokyo, 113-8421, Japan; Sportology Center, Juntendo University, Tokyo, 113-8421, Japan; Sportology Center, Juntendo University, Tokyo, 113-8421, Japan; Department of Metabolism & Endocrinology, Juntendo University, Tokyo, 113-8421, Japan; Sportology Center, Juntendo University, Tokyo, 113-8421, Japan; Sports Medicine & Sportology, Juntendo University Graduate School of Medicine, Tokyo, 113-8421, Japan; Department of Metabolism & Endocrinology, Juntendo University, Tokyo, 113-8421, Japan; Department of Metabolism & Endocrinology, Juntendo University, Tokyo, 113-8421, Japan; Department of Metabolism & Endocrinology, Juntendo University, Tokyo, 113-8421, Japan; Department of Metabolism & Endocrinology, Juntendo University, Tokyo, 113-8421, Japan; Department of Metabolism & Endocrinology, Juntendo University, Tokyo, 113-8421, Japan; Department of Metabolism & Endocrinology, Juntendo University, Tokyo, 113-8421, Japan; Department of Metabolism & Endocrinology, Juntendo University, Tokyo, 113-8421, Japan; Sportology Center, Juntendo University, Tokyo, 113-8421, Japan; Sports Medicine & Sportology, Juntendo University Graduate School of Medicine, Tokyo, 113-8421, Japan; Department of Metabolism & Endocrinology, Juntendo University, Tokyo, 113-8421, Japan; Sportology Center, Juntendo University, Tokyo, 113-8421, Japan; Department of Metabolism & Endocrinology, Juntendo University, Tokyo, 113-8421, Japan; Sportology Center, Juntendo University, Tokyo, 113-8421, Japan; Sports Medicine & Sportology, Juntendo University Graduate School of Medicine, Tokyo, 113-8421, Japan

**Keywords:** sarcopenia, handgrip strength, insulin resistance, triglyceride glucose index, type 2 diabetes

## Abstract

**Context:**

Older adults with sarcopenic obesity are at high risk for type 2 diabetes mellitus (T2DM). However, few East Asians have sarcopenic obesity. Since many East Asians have insulin resistance (IR) without obesity, it is possible that older East Asians with sarcopenia and IR might be at high risk for T2DM. However, this relationship has not been studied.

**Methods:**

This cross-sectional study included 1629 older adults aged 65 to 84 years registered in the Bunkyo Health Study. All underwent a 75-g oral glucose tolerance test and handgrip strength measurement. Participants were classified into 4 groups by possible sarcopenia (handgrip strength <28 kg in men and <18 kg in women) and IR status (triglyceride glucose [TyG] index ≥8.79 for men and ≥8.62 for women [third quartile]). Modified Poisson regression was used to estimate relative risk (RR) and 95% CIs for T2DM with adjustment for confounding factors.

**Results:**

The mean age was 73.1 ± 5.4 years. T2DM was diagnosed in 212 (13.0%) participants. After adjusting for age, sex, body mass index, use of lipid-lowering medications, hypertension, and cardiovascular disease, possible sarcopenia and IR were associated with T2DM, with their coexistence showing a notably stronger association (control: RR, 1.00 [Reference]; possible sarcopenia: RR, 1.55 [95% CI, 1.04-2.30]; IR: RR, 2.69 [95% CI, 1.99-3.65]; and IR possible sarcopenia: RR, 4.76 [95% CI, 3.34-6.79]).

**Conclusion:**

Possible sarcopenia based on low handgrip strength and IR based on the TyG index are independently associated with T2DM in older Japanese individuals. Their coexistence shows a particularly strong association with T2DM.

The increase in type 2 diabetes mellitus (T2DM) in older adults due to sarcopenia, obesity, and insulin resistance (IR) caused by the deterioration of body composition with age is a serious problem. Sarcopenic obesity is a condition in which sarcopenia [1], a decrease in muscle strength, skeletal muscle mass, and physical performance, and obesity coexist [2]. Individuals with sarcopenic obesity have a higher risk for T2DM [3], hypertension [4], reduced activities of daily living [5], cardiovascular disease (CVD) [6], and all-cause mortality [7] than individuals only with sarcopenia or obesity. Since skeletal muscle is the major structure responsible for systemic glucose uptake, low skeletal muscle volume and IR might synergistically decrease glucose uptake and impair glucose intolerance [8, 9].

IR is the essential pathology linking obesity to metabolic comorbidities. However, obesity does not necessarily cause IR. Obese individuals without IR, that is, metabolically healthy obese individuals, do not have a higher risk for T2DM, CVD, or all-cause mortality than normal-weight individuals [10, 11]. Conversely, nonobese people with IR, defined as metabolically obese and normal-weight [12], were reported to have a higher risk of T2DM and CVD than insulin-sensitive, normal-weight individuals [10], indicating that IR is more closely associated with cardiometabolic characteristics than obesity. Among older East Asians, the prevalence of obesity is low. Thus, few of them have sarcopenic obesity. However, since many East Asians are IR without being obese, it is possible that people with sarcopenia and IR might be at high risk for T2DM. How many such people exist and whether they really are at risk for T2DM has not been studied.

From these backgrounds, the purpose of the present study was to investigate the prevalence of possible sarcopenia (defined by low handgrip strength) and IR and their association with T2DM in community-dwelling older Japanese individuals, a population in which obesity is less common but IR may be prevalent. Furthermore, considering the anticipated rise in the number of older adults with T2DM, it is crucial for clinical tools to be both accessible and efficient for large-scale screening. Therefore, our study used the triglyceride glucose (TyG) index as a measure of IR, and handgrip strength as a measure of possible sarcopenia. The TyG index is advantageous as it does not require insulin levels, making it a practical option for widespread clinical application.

## Materials and Methods

### Study Design and Participants

The study individuals were all participants in the Bunkyo Health Study [13], a prospective cohort study. For this study, we performed a cross-sectional analysis using baseline data from the study. A total of 1629 individuals aged 65 to 84 years participated in the Bunkyo Health Study from November 2015 to September 2018. Exclusion criteria were the placement of a pacemaker or defibrillator and diabetes requiring insulin therapy. Study participants underwent 2 days of evaluations. We assessed cognitive function, physical performance, and muscle strength the first day. On the second day, following an overnight fast, we assessed abdominal fat distribution using magnetic resonance imaging and glucose tolerance using a 75-g oral glucose tolerance test (OGTT). The study protocol was approved by the ethics committee of Juntendo University in November 2015 (Nos. 2015078, and M15-0057). This research was conducted in accordance with the principles outlined in the Declaration of Helsinki. All participants provided written informed consent and were notified that they had the right to withdraw from the trial at any time.

### Definition of Type 2 Diabetes Mellitus

Diabetes was defined as fasting plasma glucose of 126 mg/dL or greater and/or a 2-hour glucose level of 200 mg/dL or greater during a 75-g OGTT, and glycated hemoglobin A_1c_ (HbA_1c_) greater than or equal to 6.5%, or current use of medication for T2DM.

### Calculations of Insulin Resistance Indices

The TyG index was calculated as ln[fasting triglyceride (mg/dL) × fasting glucose (mg/dL)/2] [14]. The homeostasis model assessment of insulin resistance index (HOMA-IR) was calculated as fasting plasma glucose (mg/dL) × insulin (mU/mL)/405 [15]. The Matsuda index was calculated as 10 000/√ ([fasting plasma glucose × fasting plasma insulin] [mean glucose × mean insulin] [16]. Adipose tissue insulin resistance index (Adipo-IR) was calculated as fasting insulin (µU/mL) × fasting free fatty acid (FFA) (mEq/L) [17].

### Handgrip Strength Measurement and Body Composition

We evaluated handgrip strength using a dynamometer (T. K. K. 5401, Takei Scientific Instruments) in a standing position [18]. Participants held the dynamometer at thigh level and 2 measurements were taken for each hand. The maximum grip strength value averaged across both hands was used in the analysis. Body composition was measured with bioelectrical impedance analysis (BIA) (InBody770, InBody Japan Inc). Skeletal muscle mass index (SMI) was calculated by dividing appendicular skeletal muscle mass by height squared in meters (kg/m^2^). Arm lean mass and arm fat mass were averaged for both arms. Percentage body fat (PBF) was calculated by dividing body fat mass (kg) by body weight (kg). In this study, sarcopenia was defined as low handgrip strength (<28 kg for men and <18 kg for women) and low SMI (<7.0 kg/m^2^ for men and <5.7 kg/m^2^ for women) based on the definition of the Asian Working Group for Sarcopenia (AWGS) 2019 [19]. In addition, possible sarcopenia was defined as only low handgrip strength.

### Other Measurements

Physical activity levels were evaluated using the International Physical Activity Questionnaire [20]. Blood samples were collected in the morning after an overnight fast for biochemical testing. All blood samples were tested at the commissioned clinical laboratory center (SRL Inc). Hypertension was defined as systolic blood pressure greater than or equal to 140 mm Hg, diastolic blood pressure greater than or equal to 90 mm Hg, or current use of antihypertensive medications. Dyslipidemia was defined as low-density lipoprotein cholesterol greater than or equal to 140 mg/dL, high-density lipoprotein cholesterol less than 40 mg/dL, triglycerides greater than or equal to 150 mg/dL, or current use of lipid-lowering agents. We evaluated cognitive function using the Montreal Cognitive Assessment [21]. Possible scores range from 0 to 30 points.

### Statistical Analysis

IR was defined based on the TyG index, which is widely used as an index for IR. It has the advantage of not requiring measurement of insulin levels [14, 22]. We used the third quartile of the TyG index as the cutoff point to divide participants into 2 groups: normal (<8.79 for men and < 8.62 for women) and IR (≥8.79 for men and ≥8.62 for women). Participants were divided into 4 groups according to the presence or absence of sarcopenia and IR: nonpossible sarcopenia/non-IR group (control), possible sarcopenia/non-IR group (possible sarcopenia), nonpossible sarcopenia/IR group (IR), and possible sarcopenia/IR group (IR possible sarcopenia).

Data are presented as means ± SD or number (%). Differences in means and proportions were tested with one-way analysis of variance and the chi-square test. The groups were compared using the Tukey-Kramer or Games-Howell post hoc test. In addition, we used analysis of covariance (ANCOVA) with Bonferroni correction to compare blood test data. Modified Poisson regression was used to estimate relative risk (RR) and 95% CIs for diabetes in each group with adjustment for confounding factors such as age and sex (model 1), variables in model 1 plus body mass index (BMI) (model 2) and in model 2 plus CVD, hypertension, and use of lipid-lowering medications (model 3). A *P* value of less than 5% was considered to be statistically significant. All analyses were performed using the SPSS statistical package.

## Results

### Clinical Characteristics of Each Group

The characteristics and anthropometric data of study participants are shown in Table 1 and Supplementary Table S1 [23]. The distribution of participants by group was as follows: control group, 61.0% (57.8% among men and 63.3% among women, n = 993); possible sarcopenia group, 14.4% (17.8% among men and 12.0% among women, n = 235); IR group, 20.0% (20.0% among men and 20.1% among women, n = 326), and IR possible sarcopenia group, 4.6% (4.5% among men and 4.7% among women, n = 75). The study participants in the 2 groups with sarcopenia were older than those in the control group. BMI, PBF, and arm fat mass were higher in the 2 IR groups than in the 2 non-IR groups. However, the mean BMI of the 2 IR groups was less than 25. Appendicular skeletal muscle mass and arm lean mass were lower in the 2 groups with possible sarcopenia compared to those without, and these were higher in the IR group than in the control group. SMI was lower in the possible sarcopenia group than in the control group, and lower in the 2 groups with possible sarcopenia than in the IR group. Daily physical activity level and Montreal Cognitive Assessment were lower in the 2 groups with possible sarcopenia than in the control group.

**Table 1.** Clinical characteristics of the study participants

	Control	Possible sarcopenia	IR	IR possible sarcopenia	*P*
No. of participants, n (%)	993 (61.0%)	235 (14.4%)	326 (20.0%)	75 (4.6%)	.199
Handgrip strength, kg	27.0 ± 6.9	20.5 ± 5.3*^a^*	27.7 ± 6.8*^b^*	19.4 ± 5.0*^a,c^*	<.001
Triglyceride glucose index	8.2 ± 0.3	8.2 ± 0.4	9.1 ± 0.3*^a,b^*	9.1 ± 0.3*^a,b^*	<.001
Male sex, n (%)	397 (40.0%)	122 (51.9%)	137 (42.0%)	31 (41.3%)	.011
Age, y	72.4 ± 5.2	76.2 ± 5.2*^a^*	72.3 ± 5.0*^b^*	76.5 ± 5.2*^a,c^*	<.001
Systolic blood pressure, mm Hg	135.3 ± 17.1	137.0 ± 17.7	139.5 ± 16.6*^a^*	140.5 ± 15.0	<.001
Diastolic blood pressure, mm Hg	83.8 ± 9.7	83.7 ± 9.6	86.1 ± 9.9*^a^*	85.0 ± 9.0	<.001
Smoking, n (%)					
Never	593 (59.7%)	146 (62.1%)	177 (54.5%)	47 (62.7%)	.227
Past	330 (33.2%)	72 (30.6%)	115 (35.3%)	26 (34.7%)	.709
Current	70 (7.0%)	17 (7.2%)	33 (10.2%)	2 (2.7%)	.105
Body mass index	22.4 ± 3.0	22.1 ± 3.1	24.3 ± 2.8*^ab^*	23.6 ± 2.8*^a,b^*	<.001
% Body fat	27.2 ± 7.1	28.0 ± 7.5	31.2 ± 6.5*^ab^*	32.2 ± 7.0*^a,b^*	<.001
Appendicular skeletal muscle mass, kg	22.1 ± 4.8	20.8 ± 4.3*^a^*	23.0 ± 4.7*^a,b^*	20.3 ± 4.1*^a,c^*	<.001
Skeletal muscle mass index, kg/m^2^	6.4 ± 1.0	6.2 ± 1.0*^a^*	6.7 ± 0.9*^a,b^*	6.2 ± 0.9*^c^*	<.001
Arm lean mass, kg	2.0 ± 0.6	1.9 ± 0.5*^a^*	2.2 ± 0.6*^ab^*	1.8 ± 0.5*^a,c^*	<.001
Arm fat mass, kg	1.0 ± 0.4	1.1 ± 0.4	1.3 ± 0.5*^ab^*	1.3 ± 0.4*^a,b^*	<.001
Abdominal subcutaneous fat, area cm^2^	144.8 ± 58.3	135.6 ± 55.3	167.0 ± 57.4*^a,b^*	163.6 ± 58.5*^a,b^*	<.001
Abdominal visceral fat area, cm^2^	70.5 ± 34.4	75.8 ± 36.3	102.7 ± 43.0*^a,b^*	91.0 ± 33.6*^a,b^*	<.001
Physical activity level, METs/h/wk	12.1 ± 16.3	8.6 ± 12.2*^a^*	11.5 ± 15.5	5.7 ± 8.3*^ac^*	<.001
Montreal Cognitive Assessment score	25.6 ± 2.7	23.6 ± 3.7*^a^*	25.2 ± 2.7*^b^*	23.9 ± 3.8*^a,c^*	<.001
Education, y	14.0 ± 2.4	13.8 ± 2.7	13.8 ± 2.4	13.4 ± 2.6	.131
Cardiovascular disease, n (%)	203 (20.6)	71 (30.3)	83 (25.5)	17 (22.7)	.009
Hypertension, n (%)	611 (61.5%)	163 (69.4%)	242 (74.2%)	61 (81.3%)	<.001
Dyslipidemia, n (%)	541 (54.5%)	132 (56.2%)	289 (88.7%)	62 (82.7%)	<.001
Drug use, n (%)	294 (29.6%)	88 (37.4%)	131 (40.2%)	38 (50.7%)	<.001
Type 2 diabetes, n (%)	72 (7.3%)	33 (14.0%)	74 (22.7%)	33 (44.0%)	<.001
Drug use, n (%)	57 (5.7%)	25 (10.6%)	47 (14.4%)	27 (36.0%)	<.001

Data are expressed as means ± SD and n (%). *P* values are from one-way analysis of variance or χ^2^ test.

Abbreviations: IR, insulin resistance; METs, metabolic equivalents.

*P* less than .05: *^a^*vs control, *^b^*vs possible sarcopenia, *^c^*vs IR, Tukey-Kramer or Games-Howell post hoc test.

Compared to the 2 non-IR groups, levels of triglycerides, fasting blood glucose, fasting serum insulin, homeostatic model assessment of β-cell function (HOMA-β), HbA_1c_, FFA, and Adipo-IR were significantly higher and Matsuda index and adiponectin levels were significantly lower in the 2 IR groups (Table 2). The area under the curve for glucose during OGTT and 2-hour glucose values after OGTT were significantly increased in the order of the control group, possible sarcopenia group, IR group, and IR possible sarcopenia group (see Table 2).

**Table 2.** Blood test data

	Control	Possible sarcopenia	IR	IR possible sarcopenia	*P*
Fasting plasma glucose, mg/dL	97.0 ± 0.5	98.6 ± 1.0	108.4 ± 0.8*^a,b^*	117.2 ± 1.7*^a,b,c^*	<.001
Fasting plasma insulin, µU/mL	4.5 ± 0.1	4.6 ± 0.2	6.0 ± 0.2*^a,b^*	6.5 ± .3*^a,b^*	<.001
Insulinogenic index	0.8 ± 0.0	0.7 ± 0.1	0.7 ± 0.1	0.6 ± 0.1	.537
HOMA-β	49.1 ± 0.9	48.2 ± 2.0	53.2 ± 1.7	54.6 ± 3.4	.075
Matsuda index	7.9 ± 0.1	7.5 ± 0.2	5.8 ± 0.2*^a,b^*	5.7 ± 0.4*^a,b^*	<.001
HOMA-IR	1.1 ± 0.0	1.1 ± 0.1	1.6 ± 0.0*^a.b^*	1.9 ± 0.1*^a,b,c^*	<.001
HbA_1c_, %	5.7 ± 0.0	5.8 ± 0.0	6.1 ± 0.0*^a,b^*	6.3 ± 0.1*^a,b,c^*	<.001
Triglycerides, mg/dL	76.5 ± 1.2	78.1 ± 2.6	166.8 ± 2.1*^a,b^*	157.2 ± 4.4*^a,b^*	<.001
HDL-C, mg/dL	67.4 ± 0.5	65.6 ± 1.0	55.8 ± 0.8*^a,b^*	56.4 ± 1.7*^a,b^*	<.001
LDL-C, mg/dL	121.1 ± 1.0	116.3 ± 2.0*^a^*	124.6 ± 1.7*^b^*	126.2 ± 3.5*^b^*	<.001
Free fatty acids, mEq/L	503.8 ± 6.5	541.5 ± 13.7*^a^*	558.1 ± 11.5*^a^*	586.6 ± 23.7*^a^*	<.001
Adipo-IR, mmol/L･pmol/L	17.3 ± 0.5	18.4 ± 1.1	24.7 ± 0.9*^a,b^*	27.9 ± 1.8*^a,b^*	<.001
Adiponectin, µg/m:	12.9 ± 0.2	13.9 ± 0.4*^a^*	10.2 ± 0.3*^a,b^*	10.7 ± 0.7*^a,b^*	<.001
AUC-glucose during OGTT, mg·min/dL·10^3^	18.3 ± 1.6	19.4 ± 3.4*^a^*	21.5 ± 2.9*^a,b^*	24.5 ± 6.0*^a,b,c^*	<.001
2-h glucose after OGTT, mg/dL	138.8 ± 1.9	152.5 ± 4.0*^a^*	173.8 ± 3.4*^a,b^*	218.6 ± 7.0*^a,b,c^*	<.001

Data are expressed as means ± SD. ANCOVA was used to adjust for sex, age, and BMI.

Abbreviations: Adipo-IR, adipose tissue insulin resistance; ANCOVA, analysis of covariance; AUC, area under the curve; BMI, body mass index; HbA_1c_, glycated hemoglobin A_1c_; HOMA-β, homeostatic model assessment of β-cell function; HOMA-IR, homeostasis model assessment of insulin resistance; HDL-C, high-density lipoprotein cholesterol; IR, insulin resistance; LDL-C, low-density lipoprotein cholesterol; OGTT, oral glucose tolerance test.

*P* less than .05: *^a^*vs control, *^b^*vs possible sarcopenia, *^c^*vs IR, with Bonferroni correction.

### Prevalence of Diabetes and Relative Risk for Diabetes by Group

Among the 1629 participants, 212 (13.0%) were classified as having diabetes. Consistent with glucose levels during OGTT, the prevalence of diabetes was 7.3% in the control group, 14.0% in the possible sarcopenia group, 22.7% in the IR group, and 44.0% in the IR possible sarcopenia group.

Table 3 shows the RR for diabetes by group. Model 1, which adjusted for age and sex, revealed an association between possible sarcopenia and IR, respectively, and diabetes and a strong association between IR possible sarcopenia and diabetes (control: RR, 1.00 [reference]; possible sarcopenia: RR, 1.62 [95% CI, 1.08-2.43]; IR: RR, 3.10 [95% CI, 2.31-4.16]; and IR possible sarcopenia: RR, 5.46 [95% CI, 3.80-7.84]). Even after further adjustment for medications and comorbidity (model 3), the results were similar (control: RR, 1.00 [reference]; sarcopenia: RR, 1.55 [95% CI, 1.04-2.30]; IR: RR, 2.69 [95% CI, 1.99-3.65]; and IR sarcopenia: RR, 4.76 [95% CI, 3.34-6.79]). Additionally, we performed a preliminary focused analysis on the subset of individuals with IR (Supplementary Table S2) [23], and found that the IR possible sarcopenia group had a significantly higher RR for T2DM compared to the IR control group. These data suggested that low handgrip strength is indeed associated with an increased prevalence of T2DM, independent of IR status.

**Table 3.** Associations between prevalence of type 2 diabetes and insulin resistance–possible sarcopenia based on triglyceride glucose index

Relative risk (95% CI)				
	Crude	Model 1	Model 2	Model 3
Control	1.00 (Reference)	1.00 (Reference)	1.00 (Reference)	1.00 (Reference)
Possible sarcopenia	1.94 (1.32-2.85)	1.62 (1.08-2.43)	1.72 (1.15-2.58)	1.55 (1.04-2.30)
IR	3.13 (2.32-4.22)	3.10 (2.31-4.16)	2.84 (2.08-3.88)	2.69 (1.99-3.65)
IR possible sarcopenia	6.07 (4.33-8.51)	5.46 (3.80-7.84)	5.30 (3.68-7.65)	4.76 (3.34-6.79)
Sex		0.49 (0.38-0.63)	0.52 (0.41-0.67)	0.46 (0.36-0.60)
Age (per 1 y)		1.02 (1.00-1.05)	1.02 (1.00-1.04)	1.01 (0.99-1.03)
BMI			1.06 (1.01-1.11)	1.02 (0.98-1.07)
Lipid-lowering medications				2.40 (1.85-3.12)
Hypertension				1.13 (0.83-1.55)
Cardiovascular disease				1.26 (0.98-1.63)

Model 1 adjusted for age and sex (for women relative to men). Model 2 adjusted for age, sex, and BMI. Model 3 adjusted for age, sex, BMI, use of lipid-lowering medications, hypertension, and cardiovascular disease.

Abbreviations: BMI, body mass index; IR, insulin resistance.

Subsequently, we further analyzed sarcopenia, now defined by both low SMI and low handgrip strength, in combination with IR status. This refined categorization led to the following distribution of participants: control group, 66.1% (n = 1074); sarcopenia group, 9.3% (n = 152); IR group, 21.7% (n = 353) and IR sarcopenia group, 2.9% (n = 47). Despite this redefinition of sarcopenia, the RR for diabetes remained consistent with our initial findings (model 3: control: RR, 1.00 [reference]; sarcopenia: RR, 1.84 [95% CI, 1.19-2.85]; IR: RR, 2.82 [95% CI, 2.13-3.74]; and IR sarcopenia: RR, 4.49 [95% CI, 2.93-6.87]) (Supplementary Table S3) [23].

**Figure 1.**
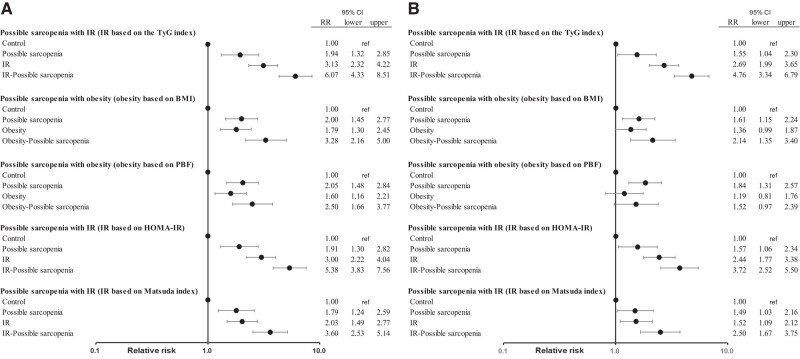
Associations between the prevalence of type 2 diabetes and IR-possible sarcopenia or obesity-possible sarcopenia. Fig. 1 shows relative risk (RR) and 95% CIs for type 2 diabetes across different groups. This figure represents the A, unadjusted, and B, fully adjusted models. The adjustments are made for age, sex, body mass index (BMI) (except for the group of obesity based on BMI), lipid-lowering medications, hypertension, and cardiovascular disease. In this analysis, possible sarcopenia was defined by low handgrip strength (handgrip strength <28 kg for men and <18 kg for women). In the corresponding groups, obesity was defined by BMI of 25 or greater or percentage body fat (PBF) of 30% or greater for men and 35% or greater for women. Insulin resistance (IR) was defined by homeostasis model assessment of insulin resistance index (HOMA-IR) of 1.64 or greater for men and 1.43 or greater for women, or Matsuda index of 4.30 or less for men and 4.58 or less for women. TyG index, triglyceride glucose index.

Additionally, in the same population and using the same method, we estimated RR and 95% CIs for diabetes using BMI, with the obesity group defined as having a BMI of 25 or greater; PBF, with the obesity group defined as having a PBF of 30% or greater for men and 35% or greater for women; HOMA-IR, with the IR group defined as having HOMA-IR 1.64 or greater for men and 1.43 or greater for women (third quartile), and Matsuda index, with the IR group defined as having a Matsuda index of 4.30 or less for men and 4.58 or less for women (first quartile), instead of the TyG index (Fig. 1). These results showed that IR is more closely associated with T2DM than the degree of obesity. Additionally, the trends of RR in each group were consistent regardless of the IR index used, and the RR value for the IR possible sarcopenia group was numerically highest when the TyG index was employed.

## Discussion

This study examined the association between IR and/or possible sarcopenia (low handgrip strength) and the prevalence of T2DM in 1629 community-dwelling Japanese older adults. We found that IR and possible sarcopenia are each independently associated with an increased prevalence of T2DM. Importantly, the prevalence is substantially higher when IR and possible sarcopenia coexist.

In this study, handgrip strength was used to define possible sarcopenia. Handgrip strength is a component for the diagnosis of sarcopenia according to AWGS 2019 [19]. Handgrip strength alone is associated with a variety of outcomes such as dementia, CV death, and all-cause mortality [18, 24, 25]. Handgrip strength is easy to assess and allows a larger population to be screened. Thus, AWGS 2019 introduces “possible sarcopenia,” defined by low muscle strength with or without reduced physical performance, which is recommended for use in primary health care and preventive services settings [19]. On the other hand, the SMI is also a diagnostic criterion for sarcopenia. However, the number of participants with IR who met the definition of reduced muscle mass was very low because the prevalence of obesity is low among older East Asians and skeletal muscle mass is strongly correlated with BMI [26]. In addition, low skeletal muscle mass alone might not be directly related to adverse events (all-cause mortality and incident disability) in older Japanese individuals [27]. Indeed, the observation in this study that the IR sarcopenia group, diagnosed with low SMI and low handgrip strength, was smaller in number and exhibited a similar association with T2DM (Supplementary Table S3) [23], supports the validity and utility of primarily using handgrip strength to evaluate sarcopenia in our study.

In this study, the TyG index was used to define IR. High TyG index has been reported to be associated with T2DM [28], hypertension [29], increased risk of CVD [30, 31], and metabolic syndrome [32, 33]. In addition, the TyG index is considered a more powerful and reliable marker than HOMA-IR for predicting metabolic syndrome [33], T2DM [34], and arterial stiffness [35]. Indeed, in this study, the use of the TyG index rather than HOMA-IR and Matsuda index as a measure of IR led to higher RR for T2DM. Furthermore, the TyG index is calculated from triglyceride and glucose levels. Since insulin concentrations, HOMA-IR, and Matsuda index are not often measured in clinical settings, the TyG index is useful for the assessment of IR in a wide range of people. However, lipid-lowering and hypoglycemic medications could affect the TyG index values by lowering fasting triglyceride and glucose levels. This could potentially lead to an underestimation of the association between the TyG index and diabetes. However, despite this potential influence, the TyG index has consistently shown an association with various outcomes, including diabetes, across different patient populations and medication statuses [36-38].

In the present study, low handgrip strength (possible sarcopenia) was independently associated with the risk of T2DM, even after accounting for IR and other confounding factors. However, there is a potential for a vicious cycle between diabetes and sarcopenia, as diabetes is known to decrease muscle mass and strength, suggesting the possibility of reverse causation in which T2DM might lead to lower handgrip strength. Previous prospective studies showed that low handgrip strength is predictive for fasting hyperinsulinemia [39] and the development of T2DM [40]. In particular, a prospective observational study using large data sets from the UK Biobank showed that low handgrip strength was a significant risk for the development of T2DM, even when adjusted for adiposity [40]. Given these findings, further longitudinal studies are warranted to better understand the complex bidirectional relationships between low handgrip strength and T2DM, thereby clarifying the mechanisms underlying these associations.

The possible sarcopenia group had lower skeletal muscle mass, lower arm lean mass, lower physical activity levels, and higher FFA concentrations than the control group, all of which are known to be risk factors for the development of T2DM [41-44]. In addition, the coexistence of IR and low handgrip strength (IR possible sarcopenia) was found to be strongly associated with an increased prevalence of diabetes. The IR possible sarcopenia group was older, had lower physical activity levels, lower skeletal muscle mass, and higher HOMA-IR than the IR alone group, all of which are also risk factors for the development of T2DM [44-47]. Thus, low handgrip strength is likely to be associated with the accumulation of clinical factors linked to the development of T2DM, thereby showing a strong association with T2DM in the possible sarcopenia and IR possible sarcopenia groups.

This study had several limitations. First, the target population of this study was relatively healthy older adults who can perform activities of daily living that lived in an urban area in Japan. The prevalence of diabetes in this study was approximately 13%, which is lower than the estimated prevalence of diabetes (“strongly suspected of suffering diabetes,” including individuals with HbA_1c_ ≥ 6.5% or currently being treated, according to the 2019 National Health and Nutrition Survey in Japan) among older adults in Japan (25.3% in men and 10.7% in women aged 60-69 years and 26.4% in men and 19.6% in women aged 70 years or older) [48]. Thus, our results might have been influenced by selection bias. Second, the TyG index cutoff value, which was used as an IR index, was not standardized with an external index. In the present study, the TyG index cutoff was the third quartile among the study participants. In previous studies, the cutoff for predicting the development of diabetes in healthy individuals was 8.8 [49] in a study with 2900 participants in China, which is very close to the present results. However, there are few reports about the TyG index, and it is not yet clear whether different cutoff values should be used for different individuals based on sex, BMI, ethnicity, or outcomes such as metabolic syndrome instead of IR [50]. However, the cutoff values we used did not deviate from several studies [51, 52], at least for the same Asian population. Finally, we cannot determine causal relationships because this is a cross-sectional study. Further observational studies are intended to clarify these issues.

In conclusion, possible sarcopenia, identified through low handgrip strength, and IR, assessed using the TyG index, were both associated with T2DM in older Japanese individuals. In particular, their coexistence was strongly associated with T2DM. Since handgrip strength and TyG index can be easily measured in clinical settings, IR possible sarcopenia might be a valuable indicator for assessing the risk of T2DM in older adults. Further studies are needed to clarify the causal relationship.

## Data Availability

Some or all data sets generated during and/or analyzed during the current study are not publicly available but are available from the corresponding author on reasonable request.

## Abbreviations

Adipo-IR: adipose tissue insulin resistance
ANCOVA: analysis of covariance
AWGS: Asian Working Group for Sarcopenia
BMI: body mass index
CVD: cardiovascular disease
FFA: free fatty acid
HbA_1c_: glycated hemoglobin A_1c_
HOMA-β: homeostatic model assessment of β-cell function
HOMA-IR: homeostasis model assessment of insulin resistance
HDL-C: high-density lipoprotein cholesterol
IR: insulin resistance
LDL-C: low-density lipoprotein cholesterol
OGTT: oral glucose tolerance test
PBF: percentage body fat
RR: relative risk
SMI: skeletal muscle mass index
T2DM: type 2 diabetes mellitus
TyG index: triglyceride glucose index
